# Coupled Charge Transfer Dynamics and Photoluminescence Quenching in Monolayer MoS_2_ Decorated with WS_2_ Quantum Dots

**DOI:** 10.1038/s41598-019-55776-6

**Published:** 2019-12-19

**Authors:** Larionette P. L. Mawlong, Abhilasha Bora, P. K. Giri

**Affiliations:** 1 Centre for Nanotechnology, Indian Institute of Technology Guwahati, Guwahati, 781039 India; 2 Department of Physics, Indian Institute of Technology Guwahati, Guwahati, 781039 India

**Keywords:** Materials science, Nanoscience and technology

## Abstract

Herein, we have investigated the tunability of the photoluminescence (PL) of the monolayer MoS_2_ (1L-MoS_2_) by decorating it with WS_2_ quantum dots (WS_2_ QD). The direct bandgap 1L-MoS_2_ and WS_2_ QDs are grown by chemical vapor deposition and liquid exfoliation methods, respectively. The room temperature PL spectrum of bare 1L-MoS_2_ is systematically quenched with its decoration with WS_2_ QDs at different concentrations. A decrease in the work function of 1L-MoS_2_ with the decoration of WS_2_ QDs was established from the Kelvin probe force microscopy analysis. A detailed quantitative analysis using the four-energy level model involving coupled charge transfer was employed to explain the redshift and the systematic decrease in the intensity of the PL peak in 1L-MoS_2_/WS_2_ QD heterostructure. The modulation of the PL in the heterostructure is attributed to the increase in the formation of negative trions through the charge transfer from WS_2_ QD to the 1L-MoS_2_ and thus making the 1L-MoS_2_ heavily n-type doped, with increase in the electron density by ~1.5 × 10^13^ cm^−2^. This study establishes the contribution of defects in the coupled charge transfer dynamics in 1L-MoS_2_, and it lays out a convenient strategy to manipulate the optical and electrical properties of 1L-MoS_2_ for various optoelectronic applications.

## Introduction

The monolayer transition metal dichalcogenides TMDs (e.g., MoS_2_, WS_2_, MoSe_2_, WSe_2_, etc.) have drawn great attention for their fascinating properties and diverse range of applications, such as transistors^1,2^, photodetectors^2–4^, light-emitting devices^5^, and sensors^6^. The strong Coulomb interactions in the atomically thin two dimensional materials create stable excitonic states even at room temperature^7,8^. Among most investigated 2D TMDs, monolayer MoS_2_ (1L-MoS_2_) has attracted significant attention due to its abundance in nature, tunable optical band gap, high chemical stability and efficient carrier generation^7–10^. An effective and convenient method to tune the optical properties of MoS_2_ is to control the charge density. To induce charge transfer to/from the 1L-MoS_2_, numerous methods were used such as chemical doping^11,12^, plasmonic hot-electron doping^13^, and electrical doping^14,15^. In the field-effect transistors (FET), application of gate bias voltage has been used to tune the charge density in the MoS_2_, however, the complex device structure fabricated on the MoS_2_ can lead to the non-uniform charge distribution and thus alter the optical measurement. Alternatively, gas molecules have also been used for carrier doping, but this method requires accurate control of the gas flow and its doping efficiency is reliant on the defect density of the material. Construction of hybrid architectures with MoS_2_ is favorable due to the excitonic nature of optical excitations in its monolayer form. Interfacing 1L-MoS_2_ with zero-dimensional semiconductor nanocrystal, also known as quantum dots (QDs) is one of the possible ways to control the optical properties of 1L-MoS_2_. The QDs have remarkable properties such as strong absorption, size-dependent energy bandgap, and high-photoluminescence. In case of a hybrid 0D/2D structure, the absorptive properties of monolayer TMD are enhanced by the QD donors which improve the optoelectronic devices, producing more efficient photodetectors and solar cells. TMD QDs such as WS_2_ QDs have gained wide interest due to their high solubility in both aqueous and non-aqueous solvents, good electrical conductivity and flexible to hybridize with other nanomaterials. Therefore, this material is highly promising for a wide range of applications. In a previous study, Li *et al*.^16^ fabricated graphene QDs/1L-MoS_2_ heterostructure (HS) and demonstrated charge transfer from graphene QDs to the 1L-MoS_2_. This charge transfer at the interface between the QD and the 1L-MoS_2_ induces competition between neutral exciton and charged exciton (trion) population resulting in the modulation in the photoluminescence (PL) of 1L-MoS_2_. Similarly, Roy *et al*.^17^ fabricated a heterostructure composed of MoSe_2_ QDs and 1L-MoS_2_ or WSe_2_ and studied the charge transfer mechanism. However, in these studies, the role of defects in PL quenching of the 1L-MoS_2_ has not been addressed. To our knowledge, there is no report on the charge transfer from WS_2_ QDs to 1L-MoS_2_ and the resulting doping and PL quenching effect. It is interesting to study the role of defects in the charge transfer dynamics in the 1L-MoS_2_ layers through PL spectroscopy and its implications for future applications. In the literature, the studies on heterostructures have been usually performed on chemically grown 2D layers, which are often multilayered and crystalline quality of layer is inferior to that grown by chemical vapor deposition (CVD) techniques.

Herein, we report a study on the tunability of the PL emission spectrum through charge transfer at the 1L-MoS_2_/WS_2_ QD HS interface. The HS was synthesized by WS_2_ QDs prepared by the liquid exfoliation method onto the CVD grown 1L-MoS_2_. The PL intensity of 1L-MoS_2_ is reduced after the formation of the 1L-MoS_2_/WS_2_ QD HS. This quenching of the PL is traced to the charge transfer from the WS_2_ QD to 1L-MoS_2_ resulting in the conversion of the neutral exciton to trion, thus making the 1L-MoS_2_ n-type doped. Additionally, the presence of defects may be another dominant factor that alters the PL emission. We show that by solving the carrier dynamics based on the coupled rate equations, we can have a better understanding of the contribution of the defects in the recombination dynamics of the hybrid structure.

## Results and Discussion

### Morphology studies

**Figure 1:**
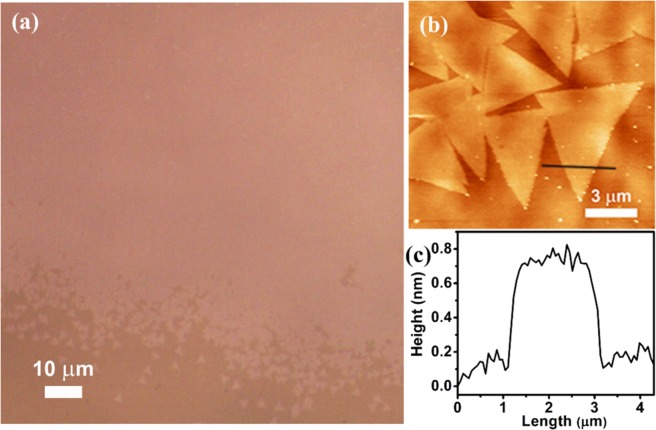
(**a**) Optical microscope image of large area monolayer MoS_2_ grown on sapphire substrate. (**b**) AFM image of triangular shaped monolayer MoS_2_ on sapphire substrate, and (**c**) AFM height profile taken along the black line in (**b**) showing a step height of ~0.7 nm confirming the monolayer MoS_2_ growth.

Figure 1(a) displays the optical image of monolayer MoS_2_ film grown with triangular-shaped MoS_2_ grains towards the edge of the sapphire substrate. These triangular shaped MoS_2_ regions merge to form a large continuous monolayer film with millimeter-scale uniformity, as evident from Fig. 1(a). The layer uniformity is evident from the small difference in contrast over the whole film. Details of the growth conditions for monolayer MoS_2_ film over a large area have been discussed in our previous work^18^. Figure 1(b) shows the AFM image of the triangular-shaped monolayer MoS_2_. It reveals that the triangular-shaped MoS_2_ have a tendency to interconnect with each other rather than overlap when they grow to form a continuous film as seen by the homogeneous color contrast, which further indicates a good uniformity. The AFM height profile taken along the black line in Fig. 1(c) indicates a thickness of ~0.7 nm, which corresponds to monolayer thickness. The AFM image of the 1L-MoS_2_/WS_2_ QD HS is shown in Fig. S1(a) (Supporting Information). The height profile of MoS_2_ layer and the QDs decorated over it clearly revealed the growth of monolayer MoS_2_ and monolayer WS_2_ QDs, as shown in Fig S1(b) (Supporting Information).

**Figure 2:**
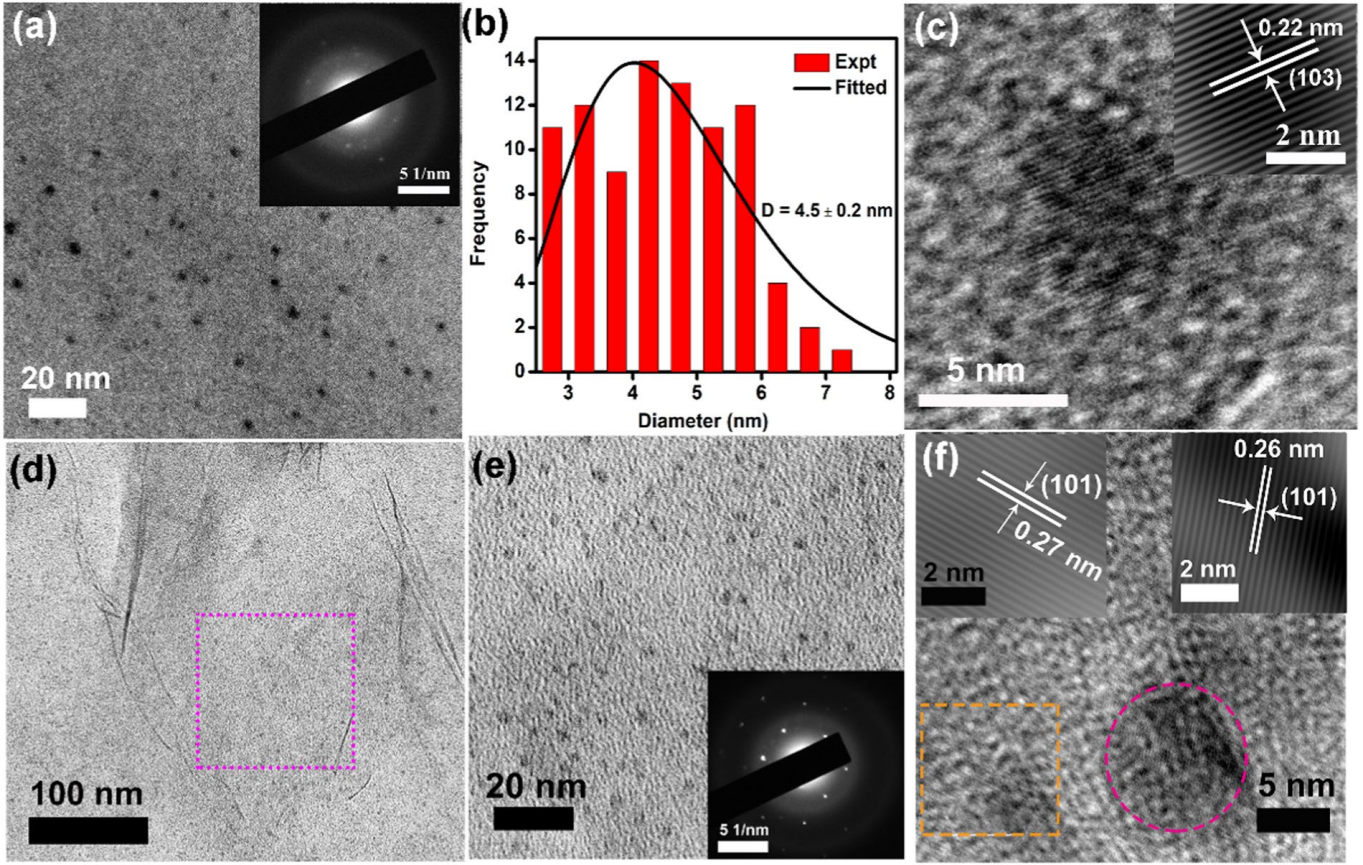
(**a**) The TEM image of WS_2_ QDs; (**b**) the size distribution of QDs with an average size of 4.5 ± 0.2 nm; (**c**) HRTEM lattice image of a WS_2_ QD; the inset shows the IFFT image of WS_2_ lattice planes. (**d**,**e**) The TEM images of uniform decoration of WS_2_ QDs on the 1L-MoS_2_ at different magnifications. The inset of 2(**e**) shows the SAED pattern with hexagonally aligned diffraction spots (for 1L-MoS_2_) and diffused rings (for WS_2_ QDs). (**f**) HRTEM lattice fringe pattern of 1L-MoS_2_/WS_2_ QDs HS. The top-left inset is the IFFT image of the region enclosed by the dotted square showing the planes corresponding to MoS_2_. The top-right inset shows the IFFT image of the area inside the dotted circle displaying the lattice fringe pattern of a WS_2_ QD.

The typical morphological and structural properties of the as-prepared WS_2_ QDs were studied using TEM. Figure 2(a) shows the TEM image of the WS_2_ QDs. The selected area electron diffraction (SAED) pattern (top right inset of Fig. 2(a)) shows the presence of diffused rings, which indicates the polycrystalline nature of the QD. The WS_2_ QDs size ranges from 3–11 nm with an average diameter of 4.5 ± 0.2 nm, as shown in Fig. 2(b). The high-resolution TEM (HRTEM) image of the WS_2_ QD (Fig. 2 (c)) displays ordered lattice fringes. The inset in Fig. 2(c) shows the inverse fast Fourier transform (IFFT) of the lattice fringes with an interplanar spacing of 0.22 nm, which corresponds to the (103) plane of WS_2_. To examine the coverage of the WS_2_ QDs on the 1L-MoS_2_, TEM imaging of the 1L-MoS_2_/WS_2_ QD HS was carried out. Figure 2(d) shows a low magnification TEM image of the QD decorated on large area 1L-MoS_2_ film. A higher magnification TEM image is depicted in Fig. 2(e), where a uniform surface coverage of WS_2_ QDs is clearly observed over the MoS_2_ layer. The corresponding SAED pattern shows the polycrystallinity of the WS_2_ QDs. In addition, hexagonally aligned diffraction spots are attributed to the (101) plane of MoS_2_ (inset of Fig. 2(e)). Thus, the as-grown 1L-MoS_2_ is highly crystalline in nature and is uniformly decorated with WS_2_ QDs. The HRTEM image of the 1L-MoS_2_/WS_2_ QD HS is displayed in Fig. 2(f), which shows distinct lattice planes. The top-left inset shows the IFFT of the atomic planes of the MoS_2_ film. The lattice d-spacing is 0.27 nm that corresponds to (101) plane of MoS_2_. Additional ordered domains are observed with a lattice spacing of 0.22 nm, which can be assigned to the (101) plane of WS_2_ (top right inset of Fig. 2(f)).

### Structural and optical analysis

**Figure 3:**
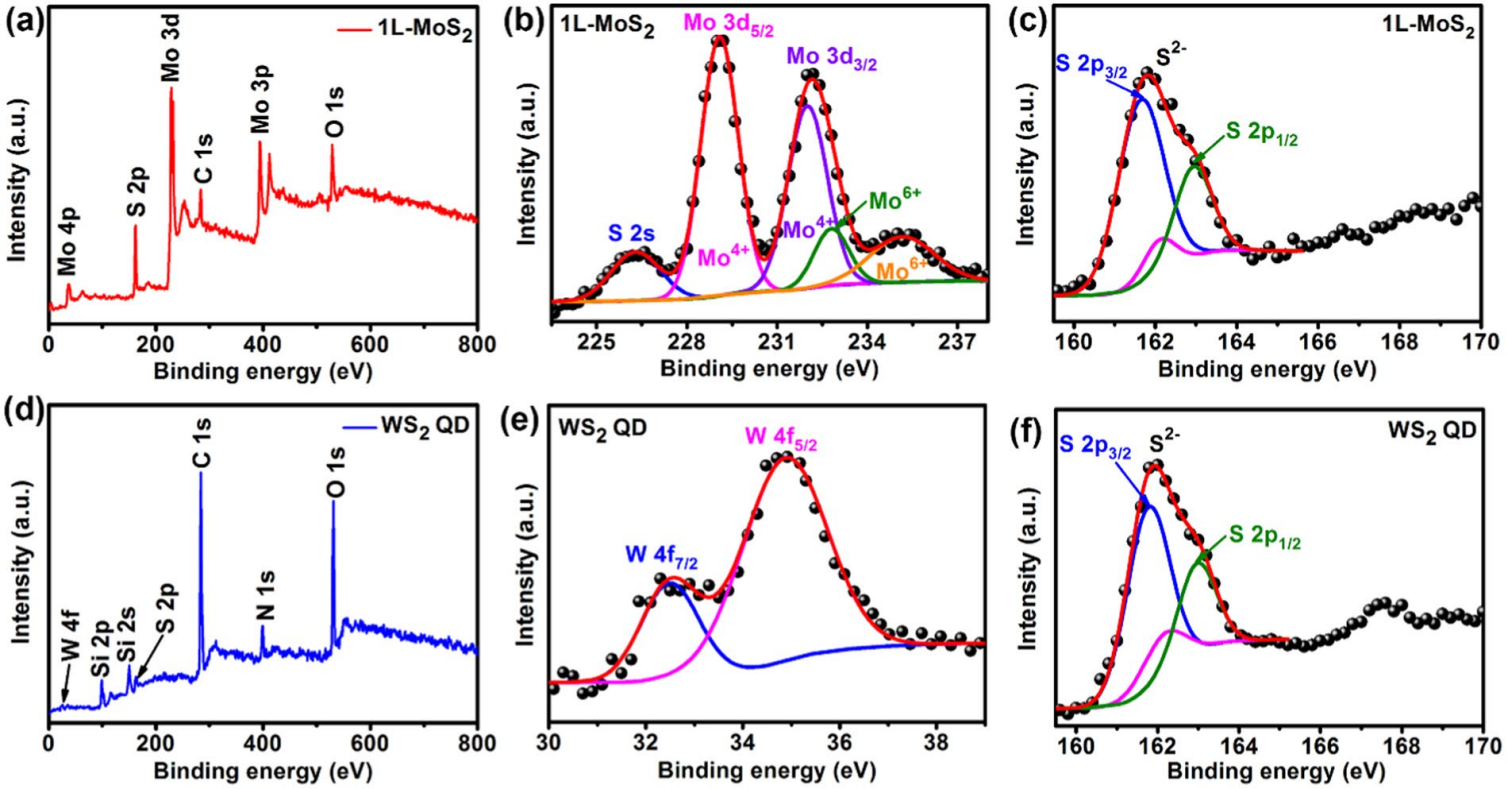
(**a**) XPS survey spectrum of 1L-MoS_2_. (**b**,**c**) Core level XPS spectra of 1L-MoS_2_ with fitting for Mo 3d, and S 2p, respectively. (**d**) XPS survey spectrum of WS_2_ QD. (**e**,**f**) Core level XPS spectra of WS_2_ QD with fitting for W 4 f and S 2p, respectively. The symbols are experimental data and the solid curves are Gaussian fittings.

The chemical composition of the 1L-MoS_2_ and WS_2_ QDs was confirmed from the XPS analysis. Figure 3 shows the XPS spectra of the core level Mo 3d, W 4 f and S 2p bands for the 1L-MoS_2_ and WS_2_ QDs samples. Figure 3(a) confirms the elemental composition of 1L-MoS_2_ with the presence of the peaks of Mo and S. In Fig. 3(b), several Mo 3d_5/2_ and 3d_3/2_ peaks fitted for Mo (3d) envelope, indicating that more than one Mo species were present. The first peak, centered at 226.4 eV, agrees well with that of the 2 s binding energy of elemental S. The strongest Mo 3d doublet peaks for 1L-MoS_2_ detected at 229.1 eV (3d_5/2_) and 232.0 eV (3d_3/2_) correspond to the +4 oxidation state of Mo, confirming the formation of MoS_2_^19^. Additional Mo peaks were observed at 232.8 eV and 235.2 eV corresponding to the oxides of Mo metal (Mo^6+^) probably due to the presence of traces of MoO_3_ in the sample after CVD growth and post-synthesis exposure to air. Figure 3(c) exhibits the S 2p XPS spectra of 1L-MoS_2_ with peaks at ∼161.8 eV (S 2p_3/2_) and ∼162.9 eV (S 2p_1/2_) corresponding to the divalent sulfide ions (S^2−^). Additionally, a peak at 162.1 eV (S 2p_3/2_) (with 8.1% spectral weight) is present that could be due to the presence of surface defects introduced during the CVD growth. These defect sites are the S vacancies as there are fewer S atoms around the Mo atoms at such sites^20^. The survey scan XPS spectrum of WS_2_ QDs shows the presence of W, S, C, N and O peaks (Fig. 3(d)). The high-resolution XPS spectrum for carbon (C 1 s) is shown in Fig. S2 (Supporting Information). The deconvoluted spectrum consists of three main components centered at 284 eV, 285.2 eV and 286.7 eV that correspond to sp^2^ hybridized carbon, sp^3^ carbon and C-O bonds, respectively^18^. It is well known that carbon dots are composed mainly of sp^3^ hybridized carbon bonds, which in our case, constitute merely of 5.8% of the high-resolution C 1 s spectrum. In contrast, the sp^2^ hybridized carbon accounts for 73.9%. These results rule out the possible presence of any carbon dots in the WS_2_ QDs samples. For the as-synthesized WS_2_ QD, the peaks at 32.5 eV and 34.8 eV are identified to be from W 4f_7/2_ and W 4f_5/2_, respectively, corresponding to the 4 + oxidation state of W, as shown in Fig. 3(c), which are consistent with those reported for 2H-WS_2_^21^. Figure 3(d) shows the S 2p XPS of the WS_2_ QD with peaks at ∼161.8 eV (S 2p_3/2_), and ∼162.9 eV (S 2p_1/2_), which are similar to that of the 1L-MoS_2_ sample. The existence of surface defects (S vacancies) in the WS_2_ QD is shown by the presence of the peak at 162.1 eV (S 2p_3/2_) (with 13.5% spectral weight), which may be created during the synthesis by liquid exfoliation method. Additionally, there is a small peak at 167.5 eV corresponding to SO_2_ which suggests the minor presence of oxidized sulfur edges.

**Figure 4:**
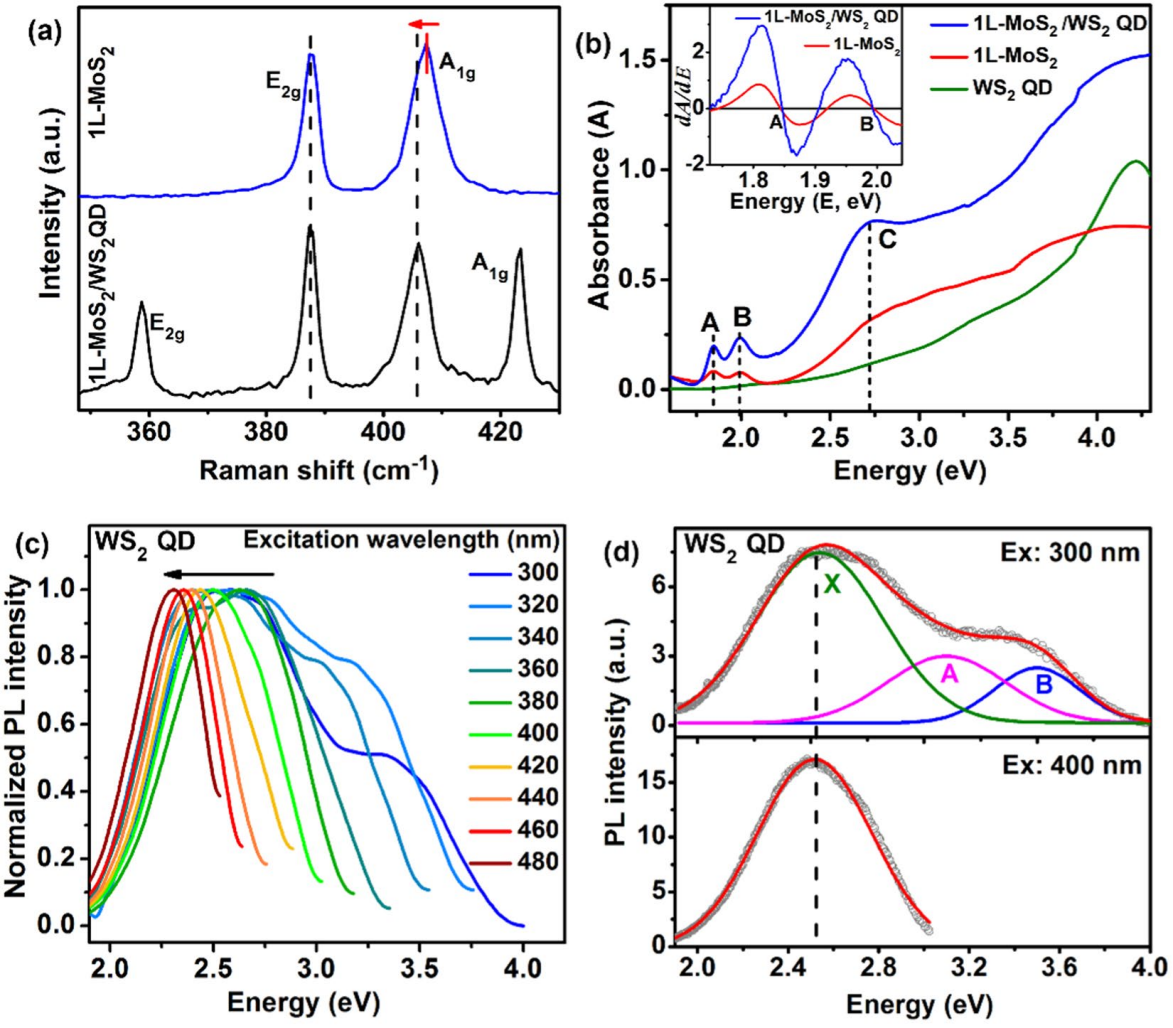
(**a**) Comparison of the Raman spectra of 1L-MoS_2_ and 1L-MoS_2_/WS_2_ QD HS (with 24 mg/L concentration of WS_2_ QD). The vertical dotted lines are indicative of no shift in the E_2g_ mode and a redshift of the A_1g_ Raman mode of MoS_2_ in the 1L-MoS_2_/WS_2_ QD HS. (**b**) Comparison of the UV-visible absorption spectra of 1L-MoS_2_, WS_2_ QDs and 1L-MoS_2_/WS_2_ QD (with 24 mg/L concentration of WS_2_ QD). A, B and C represent the characteristic excitonic absorption bands of the 1L-MoS_2_. The inset shows the first derivative of the absorption spectra of 1L-MoS_2_ and 1L-MoS_2_/WS_2_ QD to indicate any possible shift of A and B peaks. (**c**) Normalized PL emission spectra of WS_2_ QDs for various excitation wavelengths (300–480 nm). (**d**) Gaussian fitting of the PL emission spectrum for the excitation of 300 nm and 400 nm. The constituent peaks are denoted as B, A, and X excitonic emissions.

Raman spectroscopy has been widely used for the determination of the number of layers^22^, the strain, the external field and doping effects^16,23,24^ in 2D TMDs. Figure 4(a) shows the comparative Raman spectra for 1L-MoS_2_ and 1L-MoS_2_/WS_2_ QD HS at room temperature. Two characteristic Raman modes E_2g_ and A_1g_ corresponding to the in-plane vibration of Mo and S atoms and out-of-plane vibration of S atoms respectively can be clearly seen^22^. The frequency difference (Δk) between E_2g_ and A_1g_ modes has been used to identify the number of layers in MoS_2_^22^. For 1L-MoS_2_ sample, the measured Δk is ~19.6 cm^−1^ confirming the monolayer growth^22^, which is consistent with the AFM result. WS_2_ QDs also show the presence of two characteristic Raman modes E_2g_ and A_1g_ of WS_2_, which confirms the crystallinity of the QDs^25^. A comparative Raman analysis of the WS_2_ QDs and WS_2_ nanosheets shows a red shift in the E_2g_ mode and a blue shift in the A_1g_ mode in the QDs with respect to that of the nanosheets (see Fig. S3, Supporting Information). This shift in the Raman modes is attributed to the decrease in the number of layers of the WS_2_ QD compared to the WS_2_ nanosheets^25^. Interestingly, after the formation of the 1L-MoS_2_/WS_2_ QD HS, the position of the Raman modes of MoS_2_, A_1g_ is red-shifted by 1.2 cm^−1^, while that of E_2g_ is not influenced (See Table 1). This shift occurs due to the fact that the A_1g_ mode couples much more strongly with electrons than the E_2g_ mode^23^. The redshift of the A_1g_ mode indicates an effective n-type doping effect in the MoS_2_ layer due to the strong electron-phonon coupling^16^. Crystallinity of the WS_2_ QDs is further confirmed from the XRD analysis (see Fig. S4, Supporting Information) that shows a strong peak at 14.3° corresponding to the (002) plane and multiple weak peaks corresponding to (004), (101), (103), (006) and (105) lattice planes of 2H-phase of crystalline WS_2_ (JCPDS 08-0237)^26^.

**Table 1:** Summary of the Raman modes (E_2g_, A_1g_), their separation (**∆**k**)** and relative weightage of the PL peaks obtained through Gaussian deconvolution for 1L-MoS_2_ and 1L-MoS_2_/WS_2_ QD HS.

Sample	Raman modes			Relative weightage of PL peaks			
	E_2g_ (cm^−1^)	A_1g_ (cm^−1^)	∆k (cm^−1^)	B-exciton (B) (%)	A-exciton (A) (%)	Trion (A^−^) (%)	Bound exciton (X) (%)
1L-MoS_2_	387.6	407.2	19.6	13.2	53.0	23.2	10.6
1L-MoS_2_/WS_2_ QD	387.6	406.0	18.4	15.5	20.6	37.2	26.7

Figure 4(b) shows the UV-vis absorption spectra of the samples. The 1L-MoS_2_ exhibits three excitonic absorption peaks A, B and C at 1.85, 2.00 and 2.74 eV, respectively. The excitonic A and B peaks originate from the transitions between the spin-orbit split valence band and the minimum of the conduction band at the K and K′ points of the Brillouin zone^7^. The C absorption peak is assigned to the direct transition from the deep valence band to the conduction band^27^. The absorption spectrum of WS_2_ QDs (see Fig. 4(b)) shows low absorbance in the visible range and no distinct excitonic features in contrast to that of the monolayer WS_2_ reported in the literature^28^. Since the QDs are mostly monolayer, the bandgap is expected to be direct type and the optical bandgap calculated from the Tauc plot is 3.45 eV (see Fig. S5, Supporting Information), which is much higher than that of the monolayer WS_2_^28^. In case of 1L-MoS_2_/WS_2_ QD HS, three absorption peaks (A, B, C) were observed, which is consistent with the spectra of 1L-MoS_2_. A marginal enhancement in the absorbance of 1L-MoS_2_/WS_2_ QD HS compared to that of individual absorbance of 1L-MoS_2_ and WS_2_ QDs is observed in the spectral range 2.48 to 4.59 eV. The enhancement of the absorbance of the 1L-MoS_2_/WS_2_ QD heterostructure compared to that of the pristine monolayer MoS_2_ and WS_2_ QDs may be due to the combined effect of the increase in the number of layers as well as the enhanced light-material interaction in the heterostructure^29^. To determine the absorption peaks of spin-orbit split B and A excitons in the 1L-MoS_2_ and 1L-MoS_2_/WS_2_ QDs, we have taken the first derivative of the absorption spectra (see the inset of Fig. 4(b)). The A and B excitonic peaks for 1L-MoS_2_ are located at 1.844 eV and 1.990 eV, respectively. For 1L-MoS_2_/WS_2_ QD HS, there is only ~4 meV redshift in the A excitonic peak with respect to the 1L-MoS_2_. This small redshift in the A peak may be due to the n-type doping of 1L-MoS_2_ after the formation of the HS due to the charge transfer from the WS_2_ QDs to the 1L-MoS_2_. In contrast to our case of charge transfer, the shift in the excitonic peaks in the absorption spectra has been more prominent in chemically doped 1L-MoS_2_^30^.

The as-synthesized WS_2_ QDs are highly fluorescent in nature with a quantum yield (QY) of ~15%. The PL emission spectra usually depend on the wavelength of excitation due to the contribution from multiple states and size distribution^31^. Figure 4(c) displays the normalized PL emission spectra of the WS_2_ QDs for various excitation wavelengths. As the excitation wavelength is increased systematically from 300 to 480 nm, the emission peak position systematically redshift from 2.52 eV to 2.31 eV. The excitation wavelength-dependent PL shift in WS_2_ QDs is poorly understood in the literature. The broadening in PL peak usually results from the polydispersity in the WS_2_ QD size, which is attributed to the colloidal synthesis process^32,33^. To explain the broad PL spectrum in WS_2_ QDs under 300 nm excitation, we have deconvoluted the spectrum with three Gaussian peaks: the B exciton, the neutral A exciton, and the defect bound exciton X, as shown in Fig. 4(d). The A and B-excitons centered at 3.1 eV and 3.5 eV arise from the giant spin-orbit splitting of the valence band in the K-K′ point^21^. The B and A excitons arise from the splitting of the valence band at the K point due to strong spin-orbit coupling in the W atom of WS_2_^7,34^. The energy difference between these two peaks is found to be ~400 meV, which is similar to that of monolayer WS_2_^35^. The contribution from the A and B exciton is gradually reduced with increasing excitation wavelength and hence the spectrum is narrower than that with low wavelength excitation. The X band in the fitting at 2.54 eV is associated with the surface defect bound exciton X, and at higher excitation wavelength (>380 nm), the PL emission arises only from the bound exciton transition (Fig. 4(d)). Thus, the PL peak position is dictated by the excitation energy; lower the excitation energy lower will be the emission energy due to the selective excitation of energy levels. This explains the wavelength-dependent shift in the PL emission peaks in WS_2_ QDs. Note that the PL peak assignments are based on the measured bandgap and the energy band relationship: ${E}_{B}={E}_{g}-{E}_{b}+{E}_{SO}$^36^, where E_b_ is the exciton binding energy (~0.3 eV for monolayer WS_2_). E_SO_ is the energy difference arising due to splitting of the valence band due to strong spin-orbit coupling (~0.4 eV) in the W atom of WS_2_^35^. Thus, based on the measured bandgap, E_B_ is expected to be ~3.5 eV. Likewise, the A exciton peak is expected at ~3.1 eV. The deconvoluted peaks positions in Fig. 4(d) closely match with the above. Note that the defect contribution to the PL intensity is very significant in all the spectra.

**Figure 5:**
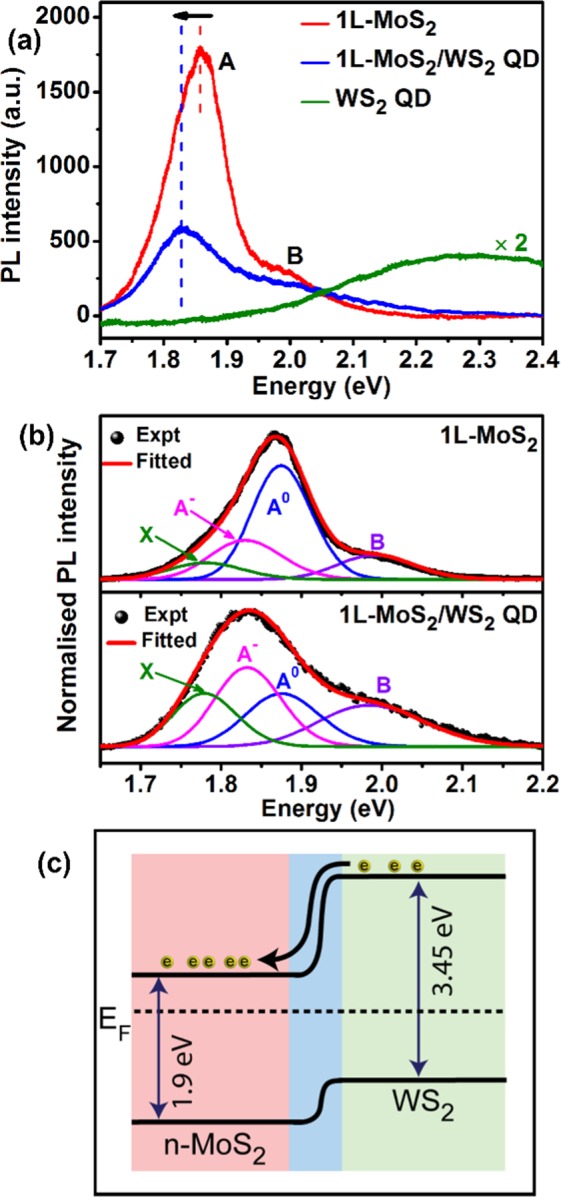
(**a**) Comparative PL spectra of pristine 1L-MoS_2_, WS_2_ QDs and 1L-MoS_2_/WS_2_ QD HS (with 24 mg/L concentration of WS_2_ QD) measured with 488 nm excitation using a micro-Raman system. (**b**) Gaussian deconvolution of PL spectra of pristine 1L-MoS_2_ and 1L-MoS_2_/WS_2_ QD HS, respectively. (**c**) Energy band diagram of the 1L-MoS_2_/WS_2_ QD heterostructure under equilibrium.

Figure 5(a) displays representative PL spectra of pristine 1L-MoS_2_, WS_2_ QD and 1L-MoS_2_/WS_2_ QD HS, measured with 488 nm laser excitation. The PL emission peak for the WS_2_ QD is broad due to the size distribution of QDs and it is much weak compared that of the 1L-MoS_2_. The PL peak position (~2.28 eV) is consistent with the result presented in Fig. 4(c)^37^. Interestingly, this peak is at a much higher energy than that of 1L-WS_2_^28^. The broadening and blue shifting of the PL peak of the WS_2_ QD originate from the quantum size effect as well as the surface defect states^31^. For 1L-MoS_2,_ we observe a PL peak at 1.86 eV with 488 nm excitation. However, after the formation of the 1L-MoS_2_/WS_2_ QD HS the PL peak position is redshifted by ~30 meV and the intensity is also partially quenched. Such a redshift and quenching of the PL is an indication of the charge transfer and n-doping effect due to the specific band alignment at the interface. This is consistent with the Raman analysis discussed earlier.

To further interpret the possible origin of the PL evolution, a deconvolution analysis was carried out by fitting each spectrum with four Gaussian peaks: the neutral exciton (A^0^), negative trion (A−), B exciton, and the defect bound exciton (X). Figure 5(b) shows the fitted PL spectra of the sample 1L-MoS_2_ and 1L-MoS_2_/WS_2_ QD HS, respectively. The A^0^ and B exciton peaks are associated with the direct bandgap transition at the K point in the Brillouin Zone, with energy split from the strong valence-band spin-orbit coupling^8^. It has been reported that the A^**−**^ trion peak arises from charged impurities in the 1L-MoS_2_ grown by a CVD method on accounts of unintentional n-type doping^38^, and the X exciton peak is assigned to the radiative recombination of bound excitons from the defect trap states^39^. Note that in the fitting process, we have fixed only the peak positions of the A^0^ (1.88 eV), B (1.98 eV), A^−^ (1.83 eV) and the X (1.78 eV) bands and the rest are kept as free parameters. With the decoration of the WS_2_ QDs, the PL spectral weight of the A^0^ exciton peak decreased from 53% to 20.6%, while that of the A^**−**^ trion peak increased from 23.2% to 37.2% (see Fig. 4(b) and Table 1). This increase in the spectral weight of the negative trion in 1L-MoS_2_/WS_2_ QD HS is due to an increase in the number of excess electrons in the 1L-MoS_2_. This is an indication that electrons are transferred from the WS_2_ QDs to the 1L-MoS_2_. Upon illumination (at 488 nm) with photon energy lesser than the bandgap (E_g_) of the WS_2_ QDs, only electrons in the defect states of the QDs absorb the photons and these electrons are excited to the conduction band. Some of these generated electrons are transferred to the 1L-MoS_2_ resulting in n-type doping, as can be understood from the schematic of the band alignment of the 1L-MoS_2_ and WS_2_ QD depicted in Fig. 5(c). DFT calculations on the MoS_2_/WS_2_ HS from previous studies show charge transfer from 1L-WS_2_ to 1L-MoS_2_^40^. The spectral weight of the defect bound excitons X increases from 10.6% to 26.7% after the formation of HS. (see Fig. 4(b) and Table 1).

**Figure 6:**
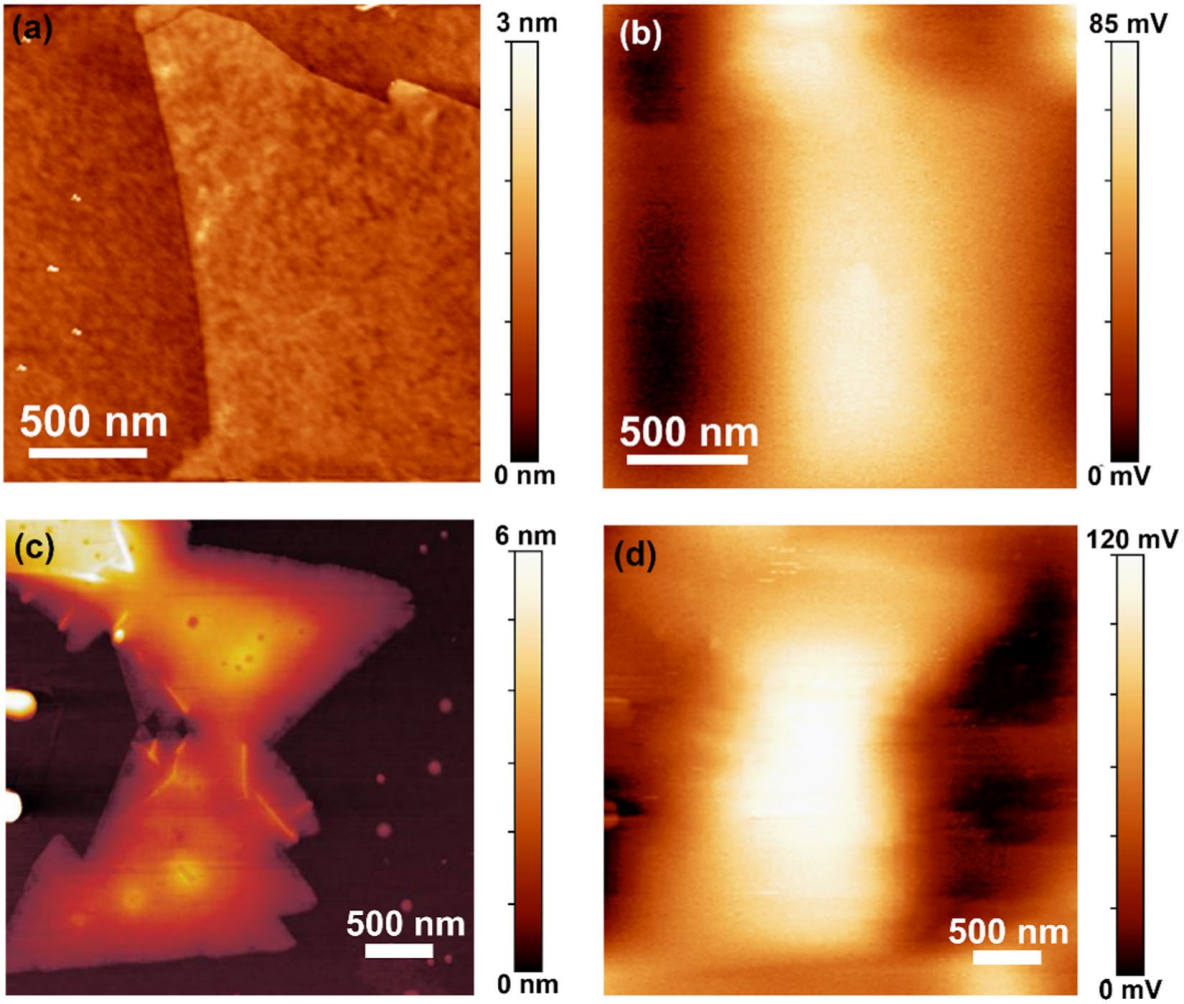
(**a**,**c**) AFM surface topography images of 1L-MoS_2_ and 1L-MoS_2_/WS_2_ QD, respectively. (**b**,**d**) The corresponding KPFM surface potential images.

To provide evidence in support of the proposed charge transfer process, the change in the work function of 1L-MoS_2_ before and after the decoration of WS_2_ QD was estimated by KPFM (Kelvin probe force microscopy). Figure 6(a,c) show the AFM topography of 1L-MoS_2_ and 1L-MoS_2_/WS_2_ QD HS, while Fig. 6(b,d) show the surface potential image of 1L-MoS_2_ and 1L-MoS_2_/WS_2_ QD HS, respectively. Before measurement, the work function of the tip (Φ_t_, in eV) was calibrated (∼4.52 eV). The overall contact potential difference (V_CPD_, in V) values of the measured samples were provided by the KPFM measurements. The measured V_CPD_ between the sample and the tip can be expressed as, e × V_CPD_ = Φ_t_ − Φ_s_, where e is the elementary charge and Φ_s_ is the work function of the sample. The contact potential difference for 1L-MoS_2_ is ~85 mV, while that for 1L-MoS_2_/WS_2_ QD HS is ~120 mV. So, the work functions of 1L- MoS_2_, Φ_1L-MoS2_ ~ 4.435 eV, which is similar to previously reported values^41^ and Φ_1L-MoS2/WS2_ ~ 4.400 eV, respectively. Thus, there is a distinct decrease in the work function of the 1L-MoS_2_/WS_2_ QD HS by 35 meV compared to 1L-MoS_2_. The reduction in the work function of the HS suggests the favorable band bending for the charge transfer from the WS_2_ QDs to the 1L-MoS_2_.

**Figure 7:**
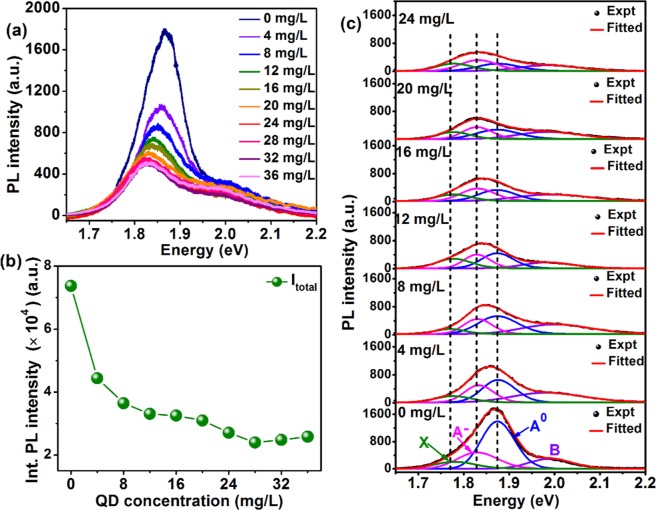
(**a**) Evolution of the PL spectra of the 1L-MoS_2_ in presence of different concentrations of WS_2_ QDs. (**b**) Integrated PL intensity of 1L-MoS_2_ as a function of the concentration of WS_2_ QDs. (**c**) Gaussian deconvolution of the PL spectra of 1L-MoS_2_ measured at different concentration of the WS_2_ QD. The PL spectra are deconvoluted with four peaks: B exciton (B), neutral exciton (A^0^), trion (A^−^), and the defect bound exciton (X).

**Figure 8:**
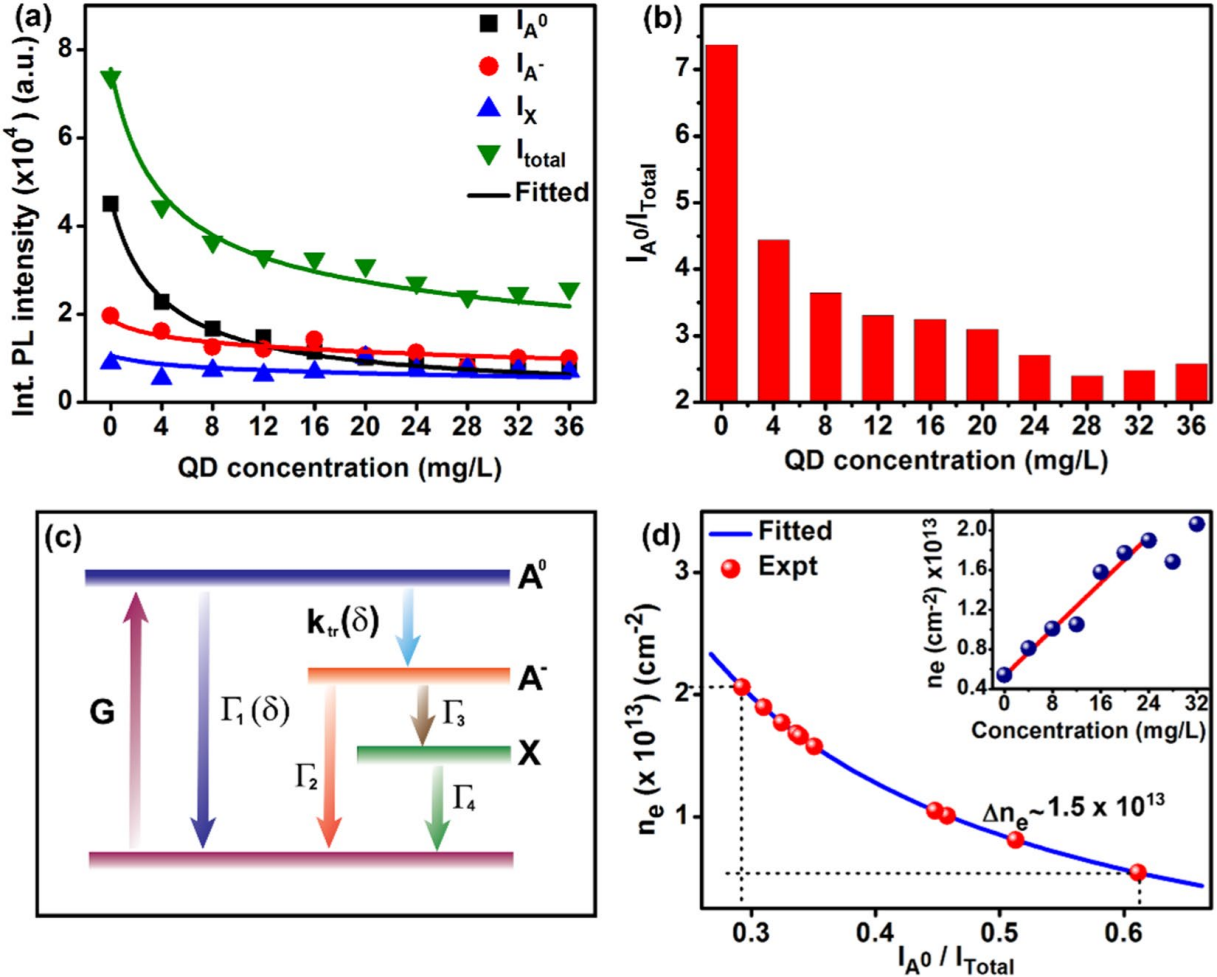
(**a**) Integrated PL intensity of neutral exciton (${{\rm{I}}}_{{{\rm{A}}}^{0}}$), trion (${{\rm{I}}}_{{{\rm{A}}}^{\mathrm{-}}}$), defect bound exciton (I_X_) and the sum (I_Total_) of ${{\rm{I}}}_{{{\rm{A}}}^{0}}$, ${{\rm{I}}}_{{{\rm{A}}}^{\mathrm{-}}}$ and I_X_ as a function of the concentration of WS_2_ QD. Symbols are the experimental data, while the solid lines are fitted data based on analytical solutions of rate equations. (**b**) The neutral exciton spectral weight (${{\rm{I}}}_{{{\rm{A}}}^{0}}$/I_Total_) as a function of the concentration of WS_2_ QD. (**c**) Schematic representation of electronic transitions through a four-level energy diagram involving the neutral exciton (${{\rm{I}}}_{{{\rm{A}}}^{0}}$), trion (${{\rm{I}}}_{{{\rm{A}}}^{\mathrm{-}}}$), defect bound exciton (I_X_) and the ground state. Other symbols are described in the text. (**d**) Calculation of electron density (n_e_) based on the law of mass action; inset shows n_e_ as a function of the concentration of WS_2_ QDs.

To further understand the change of the PL intensity of the 1L-MoS_2_ with the addition of the WS_2_ QDs (concentration 4 to 36 mg/L), PL intensity was measured for the HS system. Figure 7(a) shows the variation of the PL spectra of the 1L-MoS_2_ with different concentrations of WS_2_ QDs. The PL intensity of the 1L-MoS_2_ decreases systematically and PL peak broadens and red-shifts as the concentration of the WS_2_ QDs is increased. The total PL intensity of the 1L-MoS_2_ decreases dramatically after the formation of the 1L-MoS_2_/WS_2_ QD HS even at very low concentration (4 mg/L), as shown in Fig. 7(b). Note that attachment of WS_2_ QDs to 1L-MoS_2_ surface is limited by the specific surface area of the 1L-MoS_2_ and beyond a certain concentration, WS_2_ QDs are not directly attached to the MoS_2_ surface sites and hence further charge transfer is restricted at high concentration. To have a better understanding of the spectral changes in PL, we have considered the contribution of the neutral exciton, trion and defect bound exciton in the spectral deconvolution of PL peaks, as shown in Fig. 7(c). We believe that with increasing concentration of WS_2_ QDs, charge carrier density increases in 1L-MoS_2_. These doped electrons easily form trions and restrain the electron-hole pair recombination and as a result, the PL intensity quenches systematically and the PL peak is redshifted. Therefore, the neutral excitons are gradually converted to trions resulting in the change of the spectral weight of the individual component. It is evident from the fitting shown in Fig. 7(c), for low concentrations of the WS_2_ QD (<12 mg/L), the PL emission is dominated by the neutral exciton peak (A^0^). At higher concentration of WS_2_ QDs, the contribution of the trions becomes higher than the neutral exciton and hence induces quenching of the PL intensity and a redshift of the PL peak position. Figure 8(a) shows a plot of the integrated PL intensity of neutral excitons ${{\rm{I}}}_{{{\rm{A}}}^{0}}$, negative trions ${{\rm{I}}}_{{{\rm{A}}}^{\mathrm{-}}}$ and the bound excitons I_X_ as a function of the concentration of WS_2_ QDs. We notice that the intensity of the neutral excitons ${{\rm{I}}}_{{{\rm{A}}}^{0}}$ decreases gradually and then almost saturates at high concentration of the WS_2_ QD (>24 mg/L). However, there is a very small change in the integrated intensity of the trions. This is because the trion emission saturates after a certain doping level due to Pauli blocking effect^15^. Thus, the excess electrons that are transferred from the WS_2_ QDs to the 1L-MoS_2_ will further move to the defect trap states. It is interesting to note that despite the systematic decrease in the integrated PL intensity of A^0^ and A^−^ peaks, the defect-related X peak intensity does not decrease with doping, which is essentially due to the charge transfer from the A^−^ level to X level. In the absence of defect, one would expect an increase in trion population with increasing doping (electron) concentration, which is contrary to our experimental data. On the other hand, the total integrated PL intensity I_Total_ decreases in a similar way as that of ${{\rm{I}}}_{{{\rm{A}}}^{0}}$. Figure 8(b) shows the change of the PL spectral weight of the neutral exciton (${{\rm{I}}}_{{{\rm{A}}}^{0}}$/I_Total_) with the increase in the concentration of the WS_2_ QDs. For pristine 1L-MoS_2_ the spectral weight is ~0.61, whereas, with doping at higher concentration (>24 mg/L), the spectral weight decreases up to ~0.29. This is an indication of the transition from neutral exciton to trion with the increase in the doping.

For a quantitative understanding of the relative change in the PL intensity of the neutral exciton ${{\rm{I}}}_{{{\rm{A}}}^{0}}$, trion ${{\rm{I}}}_{{{\rm{A}}}^{\mathrm{-}}}$ and defect bound exciton I_X_, we discuss the exciton and trion relaxation dynamics with rate equations based on a four-energy level model, as shown in Fig. 8(c) ^42^. Here, G represents the generation rate of excitons, Γ_1_ and Γ_2_ represent the decay rates of the exciton and trions, respectively. k_tr_(δ) is the formation rate of trion from the exciton, which is dependent on the doping concentration (δ) of the WS_2_ QDs. To better model our experimental observation, we have assumed Γ_1_ to be dependent of δ and it is taken as proportional to doping concentration δ, without which the trion population would not decay with increasing δ, which will be evident from the solution of the rate equations discussed below. In case of high doping density, carrier-density-dependent recombination dynamics of excitons is rational and it has been reported for InGaN/GaN quantum wells^43^. Thus the dependence of Γ_1_ on δ is reasonable in the present case. The trions also decay through the defect trapping state at the rate Γ_3_. Lastly, Γ_4_ represents the decay rate of the defect bound excitons. Thus, based on the evolution of the three peaks with different doping concentrations, the electronic transitions are shown in Fig. 8(c). The corresponding rate equations for the population of neutral excitons ${{\rm{N}}}_{{{\rm{A}}}^{0}}$, trions ${{\rm{N}}}_{{{\rm{A}}}^{\mathrm{-}}}$ and the defect bound excitons N_X_ can be expressed as:

1 $$\frac{d{N}_{{A}^{0}}}{dt}=G-[{\varGamma }_{1}(\delta )+{k}_{tr}(\delta )]{N}_{{A}^{0}}$$

2 $$\frac{d{N}_{{A}^{-}}}{dt}={k}_{tr}(\delta ){N}_{{A}^{0}}-({\varGamma }_{2}+{\varGamma }_{3}){N}_{{A}^{-}}$$

3 $$\frac{d{N}_{X}}{dt}={\varGamma }_{3}{N}_{{A}^{-}}-{\varGamma }_{4}{N}_{X}$$

4 $$\,{k}_{tr}(\delta )={k}_{tr}(0)(1-s.\frac{1}{\alpha \delta +1})$$

5 $${\varGamma }_{1}(\delta )={\varGamma }_{1}(0)(1+\beta \delta )$$

where the parameter α in Eq. (4) represents the WS_2_ QD adsorption probability and β in Eq. (5) is a proportionality constant. Considering that the rate of adsorption of WS_2_ QDs obeys the Langmuir’s law, the formation rate of trions with doping concentrations can be described as k_tr_(δ) and s (∼85% for our best-fitted data) reflects the ability of charge transfer from WS_2_ QD to 1L-MoS_2_. Doping concentration δ is increased in steps for 4 mg/L in our experiment. By solving the above rate equations analytically within the framework of the four-level model (see Section S1, Supporting Information, for the full derivation), under steady-state condition, the equations reduce to

6 $${N}_{{A}^{0}}({\rm{\delta }})=\frac{G}{{\varGamma }_{1}({\rm{\delta }})+{k}_{tr}({\rm{\delta }})}$$

7 $${N}_{{A}^{-}}({\rm{\delta }})=\frac{{k}_{tr}({\rm{\delta }})}{({\varGamma }_{2}+{\varGamma }_{3})}\frac{G}{({\varGamma }_{1}({\rm{\delta }})+{k}_{tr}({\rm{\delta }}))}$$

8 $$\,{N}_{X}({\rm{\delta }})=\frac{{\varGamma }_{3}}{{\varGamma }_{4}}\frac{{k}_{tr}({\rm{\delta }})}{({\varGamma }_{2}+{\varGamma }_{3})}\frac{G}{({\varGamma }_{1}({\rm{\delta }})+{k}_{tr}({\rm{\delta }}))}$$

The steady-state PL intensities of neutral exciton (${{\rm{I}}}_{{{\rm{A}}}^{0}}$), trion (${{\rm{I}}}_{{{\rm{A}}}^{\mathrm{-}}}$) and defect bound exciton (I_X_) can be represented as follows:

9 $${I}_{{A}^{0}}({\rm{\delta }})=\frac{AG{\gamma }_{ex}}{{\varGamma }_{1}({\rm{\delta }})+{k}_{tr}({\rm{\delta }})}$$

10 $${I}_{{A}^{-}}({\rm{\delta }})=\frac{{k}_{tr}({\rm{\delta }})}{({\varGamma }_{2}+{\varGamma }_{3})}\frac{AG{\gamma }_{tr}}{({\varGamma }_{1}({\rm{\delta }})+{k}_{tr}({\rm{\delta }}))}$$

11 $$\,{I}_{X}({\rm{\delta }})=\frac{{\varGamma }_{3}}{{\varGamma }_{4}}\frac{{k}_{tr}({\rm{\delta }})}{({\varGamma }_{2}+{\varGamma }_{3})}\frac{AG{\gamma }_{X}}{({\varGamma }_{1}({\rm{\delta }})+{k}_{tr}({\rm{\delta }}))}$$

where A is the collection efficiency of luminescence, *γ*_*ex*_, *γ*_*tr*_ and *γ*_*X*_ are the radiative decay rates of neutral exciton, trion and defect bound exciton, respectively. The calculated/fitted PL intensities ${I}_{{A}^{^\circ }},\,{I}_{{A}^{-}}$ and *I*_*X*_ in Eqs. (9–11), are in excellent agreement with the experimental results, as shown in Fig. 8(a). The parameters used in this analysis are Γ_1_(0) = 0.002 ps^−1^, Γ_2_ = 0.02 ps^−1^,Γ_3_ = 0.05 ps^−1^, and k_tr_(0) = 0.5 ps^−1^, which are based on previously reported data^42,44^. We have assumed an intermediate decay rate from the defect trap state, Γ_4_ = 0.01 ps^−1^ for a good fit to the carrier recombination dynamics. The fitting parameters of AGγ_tr_/AGγ_ex_ and AGγ_X_/AGγ_ex_ to match the experimental data are 0.38 and 0.01, respectively, which implies that γ_tr_ < γ_ex_ and γ_X_ ≪ γ_ex_, consistent with their relative PL intensities observed experimentally. Note that our value of γ_tr_/γ_ex_ is nearly double of the reported value (γ_tr_/γ_ex_ = 0.15)^12^, due to the specific band alignment for favorable charge transfer and formation of trions. Due to the higher bandgap of WS_2_ QDs than that of monolayer WS_2_, the band bending is higher in our case resulting in more efficient charge transfer. Our results further imply that the defect (X) contribution to the PL evolution is smaller than the trion (A^−^) contribution. However, it is significant enough and necessary to consider it in the rate equation to match with the experimental data.

Assuming the validity of the law of mass action here, the relationship between the population of the neutral exciton (${{\rm{N}}}_{{{\rm{A}}}^{0}}$), trions (${{\rm{N}}}_{{{\rm{A}}}^{\mathrm{-}}}$) and the charge density n_e_ in the 1L-MoS_2_ is expressed as

12 $$\frac{{N}_{{A}^{0}}{n}_{e}}{{N}_{{A}^{-}}}=(\frac{16\pi {m}_{{A}^{0}}{m}_{e}}{{h}^{2}{m}_{{A}^{-}}}){k}_{B}T\,\exp (-\frac{{E}_{b}}{{k}_{B}T})$$

where h is the Planck’s constant, k_B_ is the Boltzmann constant, T is the temperature and E_b_ is the trion binding energy. The effective masses of the electron, hole, and trion are m_e_, m_h_ and ${{\rm{m}}}_{{{\rm{A}}}^{\mathrm{-}}}$, respectively. m_e_ and m_h_ are 0.35 m_0_ and 0.45 m_0_, where m_0_ is a free electron mass^15^. Therefore, the effective mass of a neutral exciton (${m}_{{A}^{^\circ }}$) and a trion (${m}_{{A}^{-}}$) can be calculated as ${m}_{{A}^{0}}$ = m_e_ + m_h_ = 0.8 m_0_, ${m}_{{A}^{-}}$ = 2m_e_ + m_h_ = 1.15 m_0_, respectively. Therefore, the calculated the PL spectral weight of the exciton can be expressed as

13 $$\frac{{I}_{{A}^{0}}}{{I}_{total}}=\frac{1}{1+\frac{{\gamma }_{tr}{N}_{{A}^{-}}}{{\gamma }_{ex}{N}_{{A}^{0}}\,}+\frac{{\gamma }_{X}{N}_{X}}{{\gamma }_{ex}{N}_{{A}^{0}}\,}}\approx \frac{1}{1+7.4\times {10}^{-14}{n}_{e}+4.4\times {10}^{-14}{n}_{e}}\approx \frac{1}{1+11.8\times {10}^{-14}{n}_{e}}$$

where I_total_ =${I}_{{A}^{0}}+{I}_{{A}^{-}}+{I}_{X}$, and the E_b_ and T are taken as 25 meV and 300 K, respectively. The γ_tr_/γ_ex_ and γ_X_/γ_ex_ values as obtained from the fitting are substituted here. Thus, the charge density n_e_ is calculated from the exciton spectral weight using Eq. (13) and is shown in Fig. 8(d). For pristine 1L-MoS_2_, the charge density is ~ 5.5 × 10^12^ cm^−2^ owing to its unintentional n-doping attributes^45^. After WS_2_ QD doping, in the saturation region, the calculated electron density of the 1L-MoS_2_/WS_2_ QD HS increases to 20.5 × 10^12^ cm^−2^. It is important to note that the difference in the electron density before and after the formation of the HS is, Δn_e_ ~ 1.5 × 10^13^ cm^−2^, which is significant. This change in the electron density signifies the approximate density of doped electrons in 1L-MoS_2_. The inset in Fig. 8(d) shows the gradual increase in the charge density n_e_ in the 1L-MoS_2_ with the increase in the WS_2_ QD concentration. Thus, these results demonstrate effective control of doping/electron density in 1L-MoS_2_ about one order of magnitude by the decoration of WS_2_ QDs. We believe that the electron density in the 2D materials can be effectively tuned by decorating with QDs of other 2D materials with high bandgap and thus, enable suitable control of the electrical and optical properties of the 2D materials, which is very significant for the ensuing applications.

## Conclusion

In conclusion, we have demonstrated the tunability in the light emission of the 1L-MoS_2_ by decorating it with the WS_2_ QD. KPFM analysis revealed a decrease in the work function of 1L-MoS_2_ with the decoration of WS_2_ QDs. Systematic quenching of the PL intensity of 1L-MoS_2_ with the decoration of WS_2_ QDs was explained on the basis of charge transfer from WS_2_ QDs to 1L-MoS_2_. A detailed analysis using coupled charge transfer among four-energy levels was employed to explain the redshift and the decrease in the PL intensity of the 1L-MoS_2_ after decoration with the WS_2_ QDs. An analytical solution to the coupled rate equations for change in the population of different excitonic emissions including bound excitonic transition was successfully employed to quantitatively understand the quenching process. The contribution of defects in the charge transfer induced quenching of PL and the carrier-density-dependent recombination dynamics of excitons were established through the quantitative analysis of the spectral evolution. Charge transfer induced increase in electron density in 1L-MoS_2_ leads to the transition of the neutral excitons to trions. The change in the electron density up to Δn_e_ ~ 1.5 × 10^13^ cm^−2^ indicates high n-type doping in the 1L-MoS_2_ by a simple decoration approach. Our results suggest an effective way to manipulate the electron density through decoration/doping technique, which is advantageous to tune the optical and electrical properties of monolayer TMDs for optoelectronic applications.

## Methods

### Synthesis of WS_2_ quantum dots

High purity WS_2_ powder (Sigma Aldrich, 99%) was dispersed in 80 ml N-methyl-2-pyrrolidinone (NMP) (Alfa Aesar, HPLC grade, 95%) and tip-sonicated using an ultrasonic homogenizer (Sonic Ruptor 250, Omni International) for 15 hours. Subsequently, the suspension was allowed to settle for 12 hours and was centrifuged for 45 minutes at 12000 rpm. The top 2/3^rd^ of the solution (supernatant) contains the WS_2_ quantum dots, while the bottom 1/3^rd^ (centrifugate) comprises of the bigger WS_2_ quantum dots and the nanosheets (See Fig. S6, Supporting Information). The excess solvent from the centrifugate was evaporated with constant stirring and the resultant residue was dispersed in Milli-Q water at various concentrations (4, 8, 12, 16, 20, 24, 28, 32, 36 mg/L) for further experiments.

### Growth of monolayer MoS_2_ by chemical vapor deposition (CVD) technique and formation of heterostructure with WS_2_ quantum dots

Monolayer MoS_2_ film was synthesized on Si/SiO_2_ and Sapphire substrates by the CVD method using a two-zone horizontal muffle furnace. 15 mg of MoO_3_ powder (99.5%, Sigma-Aldrich) and 200 mg of sulfur powder (99.95%, Sigma-Aldrich) in separate quartz boats were placed inside the 2” diameter quartz tube at the center of their respective zones for the CVD growth of MoS_2_, as reported previously^46^. The substrates were placed face down on top of the quartz mask with a circular opening and then placed on the boat containing MoO_3_. Then, the quartz tube was flushed with high purity argon gas at 300 sccm for 30 minutes prior to the growth. The sources temperatures were gradually increased from room temperature to 700 °C and 150 °C at the rates 15 and 3.5 °C/min for MoO_3_ and Sulphur, respectively, and kept at this temperature for 5 minutes at an argon flow rate of 10 sccm. Afterward, the furnace is allowed to cool down to room temperature. It was observed that the 1L-MoS_2_ film was deposited only on the portions of the substrate which were covered by the quartz mask. The unmasked regions of the substrate were found to be deposited with few-layer and multilayer MoS_2_. We observed that in both the SiO_2_/Si and sapphire substrates, large-area monolayer MoS_2_ film was grown as reported in our previous work^47^.

For the formation of the heterostructure, WS_2_ QDs were spin-coated onto the 1L-MoS_2_ and are dried before optical characterizations were carried out (see Fig. S6, Supporting Information).

### Characterization techniques

The 1L-MoS_2_ grown over various substrates, WS_2_ QDs, and their heterostructure were studied by high-resolution micro-Raman spectroscopy (LabRam HR800, Jobin Yvon). Both Raman and PL spectra were acquired sequentially from the same spot on the sample through a 100X objective lens with a spot size ∼1 μm and laser power ~1.5 mW to avoid laser-induced sample damage. The signal was then collected by a charge-coupled device (CCD) using a backscattering geometry sent through a multimode fiber grating (1800 grooves mm^−1^). Atomic force microscopy (AFM) (Cypher, Oxford Instruments) images were acquired to confirm the layer thickness of CVD-grown MoS_2_ and WS_2_ quantum dots. In order to carry out the surface potential (SP) analysis of the samples, the Kelvin probe force microscopy (KPFM) measurements were done. Conducting platinum (Pt)/iridium (Ir)-coated tips were used for KPFM studies, having the optimum frequency of operation ~72 kHz. To avoid the noise between the topographical and the surface potential measuring images, the measurements were carried out in the dual-pass lift mode. The calculation of the work function for the sample (Φ_s_) was obtained from the AFM by using Pt/Ir tips in the KPFM mode. The morphology, size and structural properties of the as-prepared WS_2_ QDs were studied by a transmission electron microscope (TEM) (JEOL-JEM 2010 operated at 200 kV). Samples for TEM analysis were prepared on a carbon-coated Cu grid of 400 mesh size (Pacific Grid, USA). TEM imaging was used to examine the decoration of WS_2_ QD on 1L-MoS_2_. For this purpose, the CVD grown 1L-MoS_2_ was transferred from the SiO_2_ substrates to carbon-coated Cu-grids. To transfer as-grown MoS_2_ film, the sample was coated with polymethylmethacrylate (PMMA) by spin coating at 1500 rpm for 60 s, and then baked at 140 °C for 10 min. The PMMA-coated sample was then treated with 6 M NaOH solution for one hour to etch out the PMMA supported MoS_2_ film, which was then repeatedly washed with DI water. Then, the film was fished out onto a Cu grid and allowed to dry at low temperature (50 °C). The PMMA was removed from the MoS_2_ film by the addition of acetone dropwise. WS_2_ QDs of the concentration 4 mg/L was then drop cast on the sample for TEM imaging. A commercial spectrophotometer (PerkinElmer, Lambda 950) was used to study the UV−vis absorption spectra of the 1L-MoS_2_/WS_2_ QD HS as well as its individual counterparts.

## Supplementary information

Supplementary information
