# Supplementary material for: Coupled Charge Transfer Dynamics and Photoluminescence Quenching in Monolayer MoS2 Decorated with WS2 Quantum Dots
Source: Sci Rep. 2019 Dec 19;9:19414. doi: 10.1038/s41598-019-55776-6 (PMC6923361; doi:10.1038/s41598-019-55776-6)
Supplement: Supplementary file 1 — Supplementary information [file 41598_2019_55776_MOESM1_ESM.docx]

**Coupled Charge Transfer Dynamics and Photoluminescence Quenching in Monolayer MoS_2_ Decorated with WS_2_ Quantum Dots**

**Larionette P. L. Mawlong^1^, Abhilasha Bora^2^ and P. K. Giri^1, 2^**^[[1]](#footnote-1)^*

*^1^Centre for Nanotechnology, Indian Institute of Technology Guwahati, Guwahati -781039, India*

*^2^Department of Physics, Indian Institute of Technology Guwahati, Guwahati -781039, India*


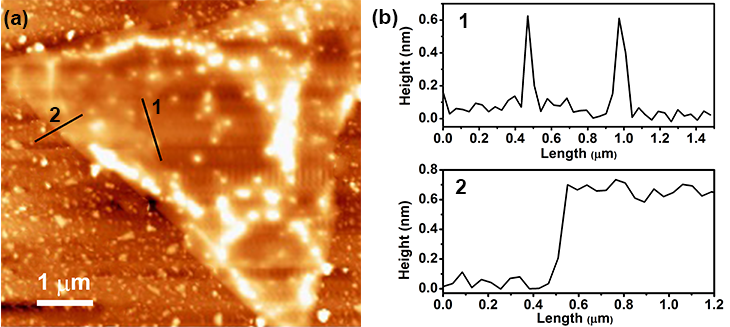


Fig. S1: (a) AFM image of triangular shaped monolayer MoS_2_ decorated with WS_2_ QDs on sapphire substrate, and (b) AFM height profile taken along the black lines (position 1 and 2) in (a) showing the height profile of WS_2_ QDs and the monolayer MoS_2_, respectively.

**
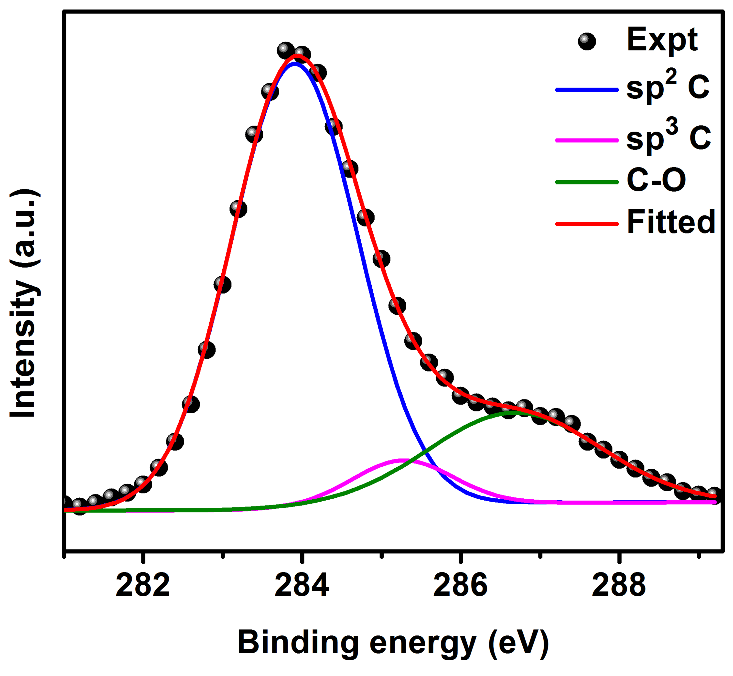
**

Fig. S2: XPS spectrum of core level C 1s in WS_2_ QD. The main source of carbon is the residual NMP in WS_2_ QDs.


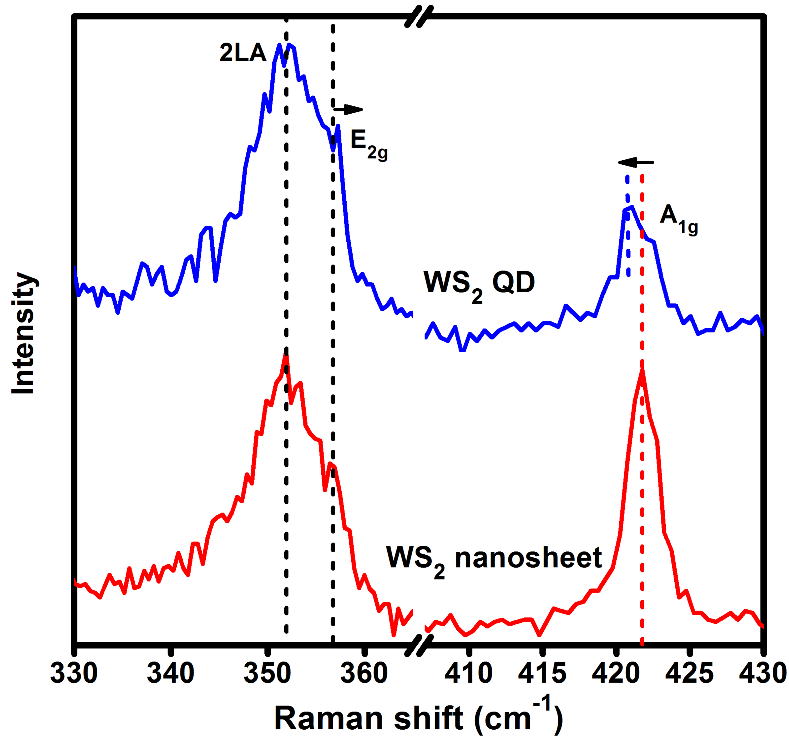


Fig. S3: Comparative Raman spectra of the WS_2_ QD and WS_2_ nanosheet depicting two Raman modes E_2g_ and A_1g_, respectively, for 532 nm excitation. The vertical dashed lines indicate relative shift of the respective modes.

**
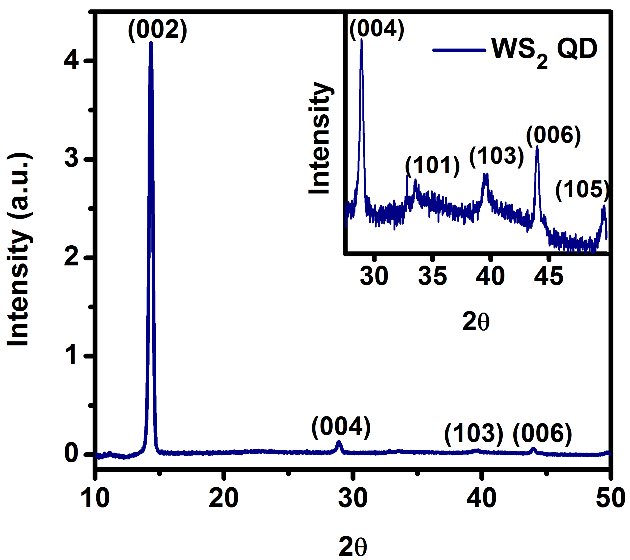
**

Fig. S4: XRD pattern of WS_2_ QDs. The inset depicts a magnified view of the XRD pattern in the range 2θ=28° to 49°.

**
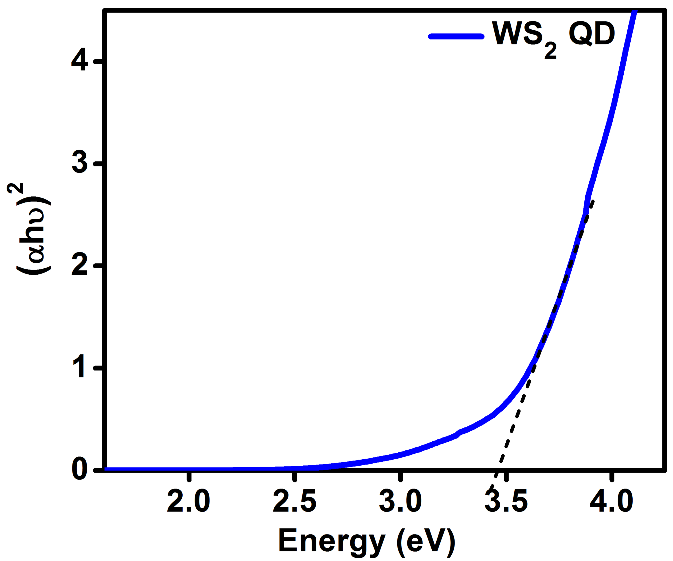
**

Fig. S5: Tauc plot for the WS_2_ QDs indicating a bandgap of 3.45 eV.

**
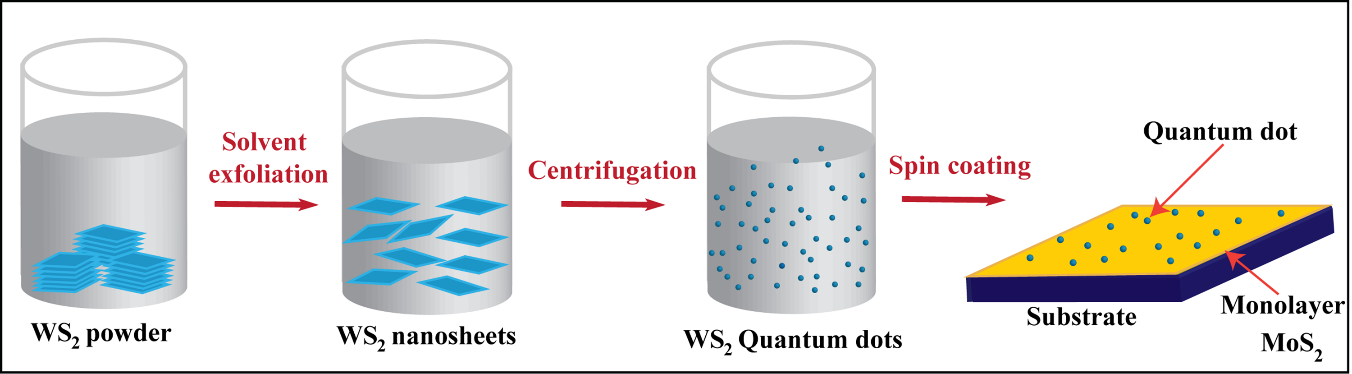
**

Fig. S6: Schematic illustration of the synthesis of WS_2_ QDs and their decoration onto 1L-MoS_2_.

**S1. Analytical solution of the charge transfer dynamics among four energy levels**

With reference to the transitions shown in Fig. 4(c), the rate equations for the population of neutral excitons (N_A_^0^), trion (N_A_^-^) and defect density (N_X_) can be expressed as

$\frac{dN_{A^{0}}}{dt}=G-[\Gamma_{1}(\delta)+k_{tr} \left( \delta\right)]N_{A^{0}}$ (S1)

$\frac{dN_{A^{-}}}{dt}=k_{tr}\left( \delta\right)N_{A^{0}}-{(\Gamma}_{2}{+\Gamma_{3})N}_{A^{-}}$ (S2)

$\frac{dN_{X}}{dt}=\Gamma_{3}N_{A^{-}}-\Gamma_{4}N_{X}$ (S3)

$k_{tr}\left( \delta\right)=k_{tr}\left( 0 \right)(1-s.\frac{1}{\alpha\delta+1})$ (S4)

$\Gamma_{1}(\delta)=\Gamma_{1}(0)(1+\beta\delta)$ (S5)

where G is the generation rate of the A exciton, $\delta$ is the concentration of WS_2_ QDs solution. Considering that the rate of adsorption of WS_2_QDs obeys the Langmuir’s law, the formation rate of trions with doping concentrations can be described as k_tr_ (δ) and s reflects the ability of charge transfer from WS_2_QD to 1L-MoS_2_. Γ_1_ and Γ_2_ are the decay rate of neutral exciton and trion, respectively and β in eq. (S5) is a proportionality constant. The time dependent population of excitons, trions and defect charges can be analytically solved as:

$N_{A^{0}}\left( \delta,t \right)=\frac{1}{\Gamma_{1}(\delta)+k_{tr}\left( \delta\right)}(G-e^{-\left( \Gamma_{1}+k_{tr}\left( \delta\right) \right)t})$ (S6)

$N_{A^{-}}\left( \delta,t \right)=\frac{k_{tr}\left( \delta\right)}{\left( \Gamma_{2}+\Gamma_{3} \right)}\frac{G}{{(\Gamma}_{1}(\delta)+k_{tr}(\delta))}\left( 1-e^{-\left( \Gamma_{2}+\Gamma_{3} \right)t} \right)-\frac{k_{tr}\left( \delta\right)}{{(\Gamma_{2}+\Gamma_{3}-(\Gamma}_{1}(\delta)+k_{tr}(\delta)){(\Gamma}_{1}(\delta)+k_{tr}(\delta))}{[e}^{-{(\Gamma}_{1}(\delta)+k_{tr}(\delta))t}-e^{-\left( \Gamma_{2}+\Gamma_{3} \right)t}]$ (S7)

$N_{X}\left( \delta, t \right)=\frac{\Gamma_{3}}{\Gamma_{4}}\frac{k_{tr}\left( \delta\right)}{{(\Gamma}_{2}+\Gamma_{3})}\frac{G}{{(\Gamma}_{1}(\delta)+k_{tr}(\delta))}\left( 1-e^{-\Gamma_{4}t} \right)-\frac{\Gamma_{3}k_{tr}\left( \delta\right)}{{{(\Gamma}_{4}-(\Gamma}_{2}+\Gamma_{3}))}\frac{G}{{(\Gamma}_{2}+\Gamma_{3}){(\Gamma}_{1}(\delta)+k_{tr}(\delta))}\left[ e^{-{(\Gamma}_{2}+\Gamma_{3})t}-e^{-\Gamma_{4}t} \right]-\frac{\Gamma_{3}k_{tr}\left( \delta\right)}{{(\Gamma}_{2}+\Gamma_{3}-{(\Gamma}_{1}(\delta)+k_{tr}(\delta))){(\Gamma}_{1}(\delta)+k_{tr}(\delta))}[\frac{1}{{(\Gamma}_{1}(\delta)+k_{tr}(\delta))}\left( e^{-\Gamma_{4}t}-e^{-{(\Gamma_{4}+\Gamma}_{1}(\delta)+k_{tr}(\delta))t} \right)-\frac{1}{{(\Gamma}_{2}+\Gamma_{3})}\left( e^{-\Gamma_{4}t}-e^{-{(\Gamma}_{2}+\Gamma_{3}+\Gamma_{4})t} \right)$ (S8)

Under steady state condition, the above equations reduces to

$N_{A^{0}}\left( \delta\right)=\frac{G}{\Gamma_{1}(\delta)+k_{tr}(\delta)}$ (S9)

$N_{A^{-}}\left( \delta\right)=\frac{k_{tr}(\delta)}{(\Gamma_{2}+\Gamma_{3})}\frac{G}{{(\Gamma}_{1}(\delta)+k_{tr}(\delta))}=\frac{k_{tr}(n)}{(\Gamma_{2}+\Gamma_{3})} N_{A^{0}}\left( \delta\right)$ (S10)

$N_{X}\left( \delta\right)=\frac{\Gamma_{3}}{\Gamma_{4}}\frac{k_{tr}\left( \delta\right)}{{(\Gamma}_{2}+\Gamma_{3})}\frac{G}{{(\Gamma}_{1}(\delta)+k_{tr}(\delta))}= \frac{\Gamma_{3}}{\Gamma_{4}} N_{A^{-}}\left( \delta\right)$ (S11)

$\frac{N_{X}}{N_{A^{0}}}=\frac{N_{X}}{N_{A^{-}}}\times\frac{N_{A^{-}}}{N_{A^{0}}}$ (S12)

1. * Corresponding author, email: [giri@iitg.ac.in](mailto:giri@iitg.ac.in) [↑](#footnote-ref-1)
